# Clinical Outcomes of Large-Bore Aspiration Thrombectomy for Tumor Pulmonary Embolism in Renal Cell Carcinoma

**DOI:** 10.1016/j.jaccas.2025.105358

**Published:** 2025-10-08

**Authors:** Anand Mulji, Tony Rizk, Yara Younan, Stephen Stringfellow, Antony Gayed

**Affiliations:** Division of Vascular Interventional Radiology, Medical University of South Carolina, Charleston, South Carolina, USA

**Keywords:** cancer, pulmonary circulation, thrombus

## Abstract

We present a 4-year longitudinal follow-up of a previously reported case involving a 66-year-old man with renal cell carcinoma who underwent radical nephrectomy, complicated by a massive pulmonary artery tumor embolism treated with large-bore aspiration thrombectomy. Serial clinical evaluations and imaging over 4 years have shown no evidence of disease recurrence following the initial intervention. This case underscores the evolving role of emergent large-bore aspiration thrombectomy in oncologic settings, not only as a therapeutic intervention but also for its diagnostic utility in enabling histopathologic characterization and guiding oncologic management.

**Take-Home Messages:**

Large-bore aspiration thrombectomy may offer long-term disease control in cases of tumor pulmonary embolism. This procedure can aid diagnosis and inform oncologic treatment planning through tissue acquisition.

## Graphical Abstract

**Figure:**
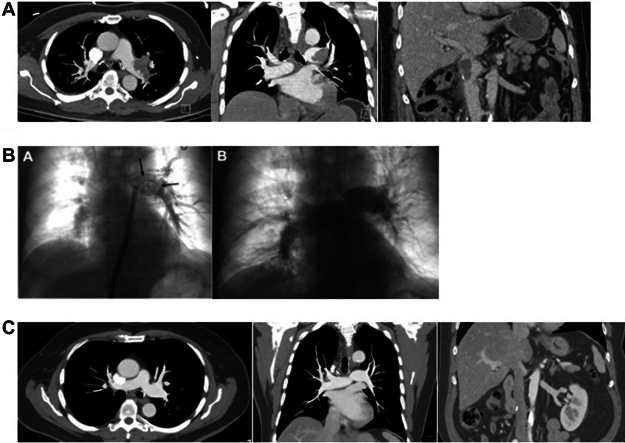

## Case Summary

A 66-year-old man presented with hematuria, anemia, and unintentional weight loss. Imaging revealed an 11-cm right renal mass with tumor extension into the right renal vein and a nonocclusive thrombus extending 4.7 cm into the infrahepatic inferior vena cava (IVC). The patient elected to undergo single-stage open right radical nephrectomy with planned IVC tumor thrombectomy. Intraoperatively, the patient experienced acute hemodynamic decompensation, and transesophageal echocardiography demonstrated a right heart thrombus in transit, which subsequently embolized into the pulmonary arterial circulation. Given the emergent circumstances, surgical IVC thrombectomy was deferred, and the nephrectomy was completed by stapling across the tumor-involved renal vein–IVC confluence. Due to the complexity of the case and surgical limitations, large-bore endovascular aspiration thrombectomy was performed the following day, targeting both the IVC and pulmonary embolism. Aspiration thrombectomy of the main and left pulmonary arteries resulted in successful extraction of a 15-cm tumor thrombus, confirmed on a histopathologic analysis. Postprocedural pulmonary angiography demonstrated resolution of the proximal embolus with improved hemodynamics. Intravenous anticoagulation was initiated on postoperative day 1, and transesophageal echocardiography revealed normal cardiac chamber sizes and estimated right atrial pressure. The patient was subsequently transferred out of the intensive care unit, recovered to his baseline functional status, and was discharged in stable condition.

**Take-Home Message**

• These cases underscore the value of large-bore aspiration thrombectomy in achieving both immediate stabilization and meaningful diagnostic insight in cancer-associated thromboembolism, ultimately informing long-term oncologic strategies.

**Figure 1:**
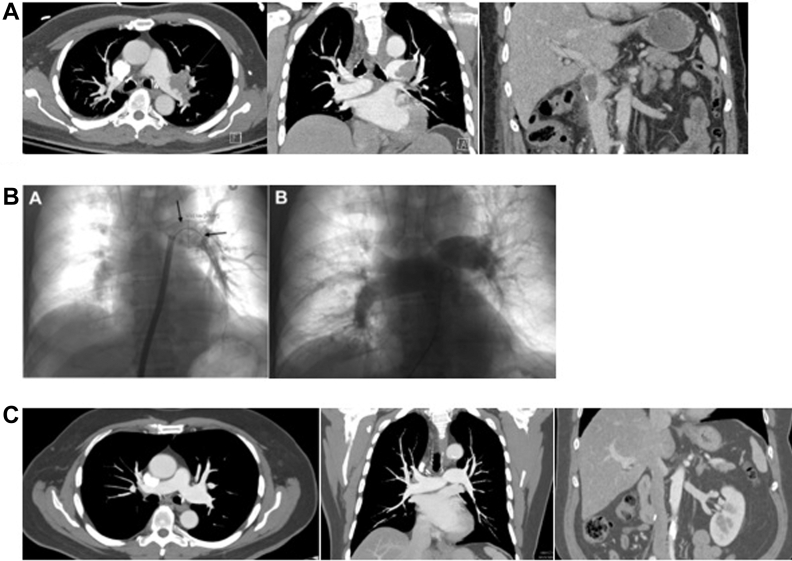
Preprocedural CT, Intraprocedural Angiography, and Postprocedural CT Images of Pulmonary Arterial Tumor Thromboembolism (A) (left to right) Axial and coronal contrast-enhanced computed tomography (CT) chest demonstrating near-occlusive left pulmonary artery thrombus. Coronal contrast-enhanced CT abdomen and pelvis demonstrating infrahepatic inferior vena cava thrombus. (B) (left to right) Intraprocedural pulmonary angiograms before and after thrombectomy demonstrating near-occlusive left pulmonary artery filling defect (arrows), which resolved with thrombectomy. (C) (left to right) Most recent follow-up axial and coronal contrast-enhanced CT chest demonstrating patent left pulmonary artery. Coronal contrast-enhanced CT abdomen and pelvis demonstrating patent infrahepatic inferior vena.

In 2021, he completed systemic therapy with pembrolizumab and axitinib. In addition, he has since undergone 28 contrast-enhanced CT examinations, including 14 CTs of the abdomen and pelvis, 13 CTs of the chest, and 1 CT pulmonary angiogram (Figure 1). None have demonstrated local disease recurrence or distant metastasis. The patient remains on indefinite anticoagulation with apixaban. Clinically, he continues to be asymptomatic without functional limitations.

## Discussion

Several case reports have described the use of large-bore aspiration thrombectomy in patients with known malignancies, in both emergent and nonemergent settings. One case involved a patient with a sarcomatoid variant of urothelial carcinoma and thrombus extending into the left renal vein and IVC, who presented with sudden-onset dyspnea. Imaging revealed bilateral segmental pulmonary emboli and a tumor thrombus in transit within the right atrium.^1^ In another case, a patient with cirrhosis presented with acute respiratory decompensation; imaging revealed previously undiagnosed hepatocellular carcinoma (HCC) and a large right atrial thrombus.^2^ In both instances, large-bore aspiration thrombectomy resulted in hemodynamic and respiratory stabilization. In the case of undiagnosed HCC, definitive diagnosis was achieved via histologic analysis of the aspirated thrombus. In another illustrative case, a patient with known HCC who had undergone surgical resection was managed conservatively with anticoagulation following a postoperative pulmonary embolism. Ten months later, the patient presented with diffuse pulmonary metastases and increased clot burden. Large-bore aspiration revealed tumor infiltration in 20% of the embolus, confirmed by immunohistochemistry to be consistent with the patient's primary HCC.^3^

## Funding Support and Author Disclosures

Dr Gayed has served as a Medtronic consultant and received grant from Bayer Research. The other authors have reported that they have no relationships relevant to the contents of this paper to disclose.
